# Transcriptomic and metabolomic analyses revealed regulation mechanism of mixotrophic *Cylindrotheca* sp. glycerol utilization and biomass promotion

**DOI:** 10.1186/s13068-023-02338-8

**Published:** 2023-05-19

**Authors:** Song Wang, Xiyi Zhou, Sha Wu, Mengkai Zhao, Zhangli Hu

**Affiliations:** 1 Guangdong Technology Research Center for Marine Algal Bioengineering; Guangdong Provincial Key Laboratory for Plant Epigenetics; Shenzhen Engineering Laboratory for Marine Algal Biotechnology; Longhua Innovation Institute for Biotechnology; College of Life Sciences and Oceanography, Shenzhen University, Shenzhen, 518060 China; 2 College of Physics and Optoelectronic Engineering, Shenzhen University, Shenzhen, 518060 China

**Keywords:** Diatom, *Cylindrotheca*, Mixotrophy, Transcriptome, Metabolome, Biomass, Fucoxanthin

## Abstract

**Background:**

Diatoms have been viewed as ideal cell factories for production of some high-value bioactive metabolites, such as fucoxanthin, but their applications are restrained by limited biomass yield. Mixotrophy, by using both CO_2_ and organic carbon source, is believed effective to crack the bottleneck of biomass accumulation and achieve a sustainable bioproduct supply.

**Results:**

Glycerol, among tested carbon sources, was proved as the sole that could significantly promote growth of *Cylindrotheca* sp. with illumination, a so-called growth pattern, mixotrophy. Biomass and fucoxanthin yields of *Cylindrotheca* sp., grown in medium with glycerol (2 g L^−1^), was increased by 52% and 29%, respectively, as compared to the autotrophic culture (control) without compromise in photosynthetic performance. As *Cylindrotheca* sp. was unable to use glycerol without light, a time-series transcriptomic analysis was carried out to elucidate the light regulation on glycerol utilization. Among the genes participating in glycerol utilization, *GPDH1*, *TIM1* and *GAPDH1*, showed the highest dependence on light. Their expressions decreased dramatically when the alga was transferred from light into darkness. Despite the reduced glycerol uptake in the dark, expressions of genes associating with pyrimidine metabolism and DNA replication were upregulated when *Cylindrotheca* sp. was cultured mixotrophically. Comparative transcriptomic and metabolomic analyses revealed amino acids and aminoacyl-tRNA metabolisms were enhanced at different timepoints of diurnal cycles in mixotrophic *Cylindrotheca* sp., as compared to the control.

**Conclusions:**

Conclusively, this study not only provides an alternative for large-scale cultivation of *Cylindrotheca*, but also pinpoints the limiting enzymes subject to further metabolic manipulation. Most importantly, the novel insights in this study should aid to understand the mechanism of biomass promotion in mixotrophic *Cylindrotheca* sp.

**Supplementary Information:**

The online version contains supplementary material available at 10.1186/s13068-023-02338-8.

## Background

For a long period of time, the contributions of diatoms to primary production (up to 40% in the ocean and 20% on the planet) via photosynthesis and their impacts on carbon and silica cycles have been well acknowledged and intensively studied [1–4]. Due to the abilities to produce a wide range of bioactive substances and nanomaterials, diatoms are emerging as excellent cell factories for biosyntheses of high-value bioproducts [5]. Among diatom-derived metabolites, fucoxanthin, eicosapentaenoic acid (EPA) as well as chrysolaminarin, owing to their potent bioactivities, have received the most attention [5–7]. The morphological diversity of diatoms is shown from their unique and intricate structures of the silicified cell wall, also termed as frustule, which are developed as drug delivery systems [8]. Furthermore, diatoms can play important roles in remediation of wastewater by efficiently removing nutrients and pollutants as well as in aquaculture as feeds [9–11]. Nevertheless, given the significance summarized above, commercial applications of diatom-derived products lag behind those from other species of economic importance, such as astaxanthin from *Haematococcus* [12]. This can be explained by the limited biomass yield of diatoms and only few cases of large-scale cultivation have been reported so far [13]. The unsatisfactory performance of diatom biomass accumulation has been recognized as the bottleneck hindering its commercialization [6, 14]. A maximum concentration of > 1 g·L^−1^ (dry weight) is considered favorable for mass production of diatom, but this can be quite challenging in autotrophic culture with CO_2_ as the sole carbon source [13–16]. Therefore, an alternative approach, which can significantly promote biomass and metabolites productivity of diatom, is required to meet an ever-increasing global demand.

Strategies, including optimization of medium recipe and environmental conditions as well as genetic engineering, were employed to improve biomass and bioproducts yields in diatoms [17–19]. The application of mixotrophy, by using external organic carbon sources, shows advantages over other trophic modes, as autotrophy and heterotrophy are subject to light availability and reduced photosynthesis-related metabolites, respectively [20, 21]. Moreover, there are only few species of diatoms that are able to use organic carbon sources without light [22]. As a consequence, mixotrophic cultivation, by assimilating organic carbon with the presence of light, has been considered as the trophic mode that combines both autotrophy and heterotrophy and capable to boost biomass and metabolites yields [23]. Studies, regarding mixotrophic cultivation of microalgae, were recently reviewed and the superiority of mixotrophy in promoting biomass and bioproduct accumulation was proved [6, 14, 23]. As summarized by Marella et al., mixotrophic cultivation was only applied to a limited number of species, among which *Phaeodactylum tricornutum*, as a model species, was intensively studied [6]. Glycerol was selected as the most commonly used organic carbon source in diatom mixotrophic cultivation and its promoting effect on *P. tricornutum* biomass accumulation was reported in previous studies [24]. However, changes in photosynthetic performance in mixotrophy remain controversial. Villanova et al. noted that *P. tricornutum*, grown mixotrophically with glycerol, exhibited faster growth and increased lipid accumulation without obvious changes in photosynthetic parameters in terms of quantum yield, nonphotochemical quenching (NPQ) and electron transport rate (ETR) [24]. Nevertheless, in another analogous study photosynthetic pigment and activity of *P. tricornutum* were significantly suppressed in mixotrophic culture [25].

With more diatom genomic sequence available and assistance of transcriptomic and metabolomic analyses, genetic background of diatom becomes clearer [26, 27]. The pathway of glycerol utilization was proposed, which enabled elucidation of the mechanism behind the promoting effect [24, 28]. Glycerol can enter cells by direct diffusion and then be converted to glyceraldehyde-3-phosphate by several key enzymes including glycerol kinase (GK), glycerol-3-phosphate dehydrogenase [NAD (+)] (GPDH) as well as triosephosphate isomerase (TIM) [28]. Metabolic flux, via glycolysis, eventually enters tricarboxylic acid (TCA) cycle, where energy and carbon skeleton can be generated to support other metabolisms [28]. In contrast to the relatively clear glycerol assimilation pathway, the knowledge, regarding the pathways where the flux is guided after TCA cycle, remains fragmentary. One explanation was metabolic flux in lipid synthesis was improved in mixotrophic *P. tricornutum* [24]. Of particular interest is *Cylindrotheca* sp. fails to metabolize glycerol without the presence of light. It can be, thus, expected a light-independent glycerol metabolism holds the potential of further improving biomass accumulation. However, the regulation of light on glycerol assimilation is still elusive.

In our previous study, cultivation of *Cylindrotheca closterium* was tested in a kind of vertical bag photobioreactors (PBRs) with a high potential and expandability for mass production [16]. *C. closterium* in the bag PBRs could accumulate as much as 25.5 mg g^−1^ fucoxanthin in biomass (dry weight) and was considered the few excellent species with a fucoxanthin content higher than 20 mg g^−1^ [7, 16]. Furthermore, *Cylindrotheca* was reported to achieve EPA content of up to 28.9 mg g^−1^ [18]. The outstanding auto-settleability of *Cylindrotheca* make it even more competitive as a potential cell factory for production of valuable metabolites. In this study, *Cylindrotheca* sp. was cultivated mixotrophically with glycerol as organic carbon source. Growth, biomass accumulation, pigment profiles and quantum yields of photosystem II in mixotrophic culture were characterized in comparison to autotrophic culture. A time-series transcriptomic analysis focusing on the transition from light into darkness was performed in both trophic modes to reveal the regulation of light on glycerol utilization. Additionally, transcriptomic and metabolomic analyses provided novel insights into the mechanism of biomass promotion, which involved a coordination of multiple pathways.

## Results

### Mixotrophy promoted growth, biomass accumulation and fucoxanthin yield

**Fig. 1:**
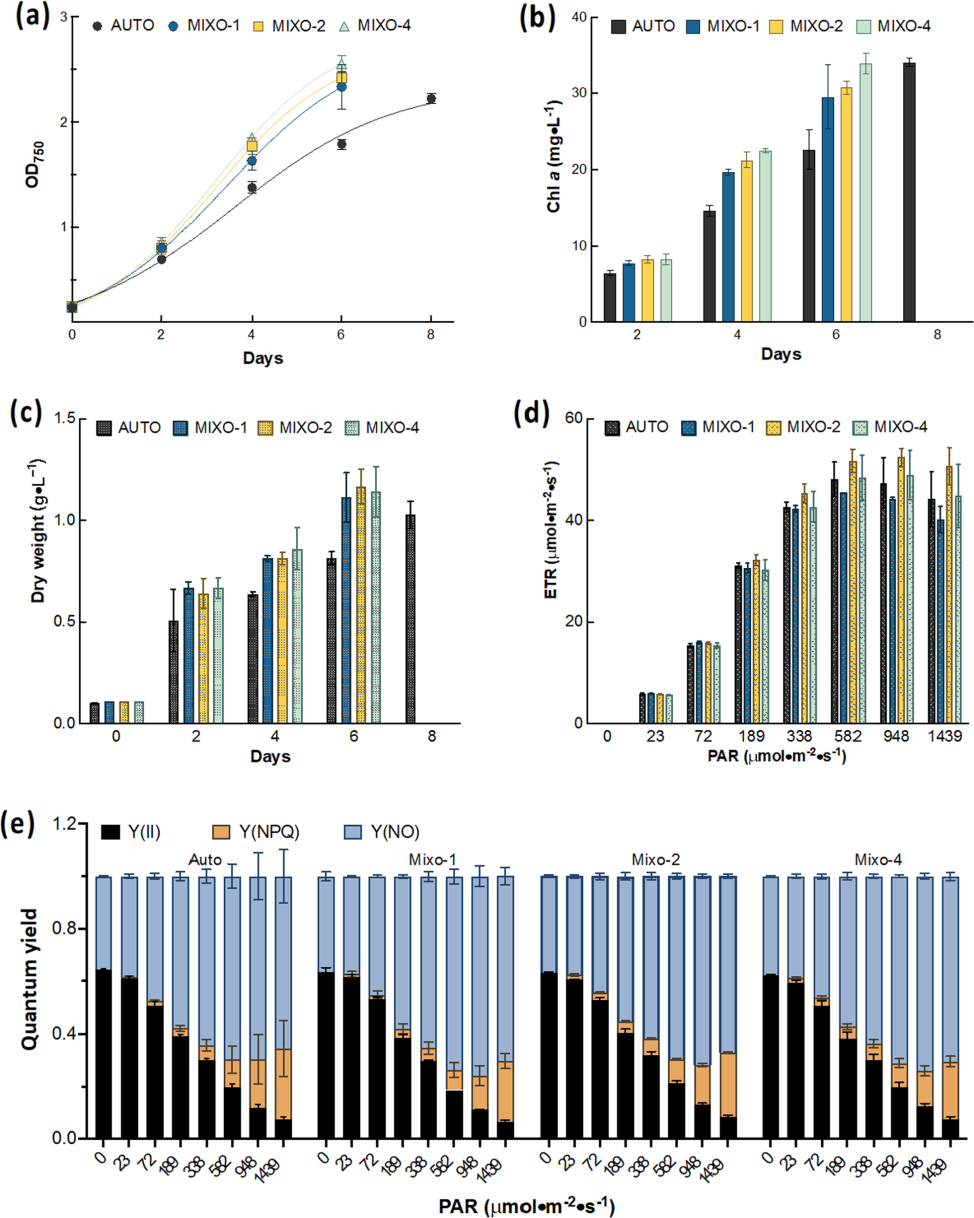
Mixotrophy with glycerol can promote growth and biomass accumulation of *Cylindrotheca* sp. **a** Growth kinetics of *Cylindrotheca* sp. in autotrophic (AUTO) and mixotrophic culture (MIXO); **b**, **c** kinetics of chlorophyll a concentration and biomass accumulation in dry weight; **d** kinetics of electron transport rate (ETR); **e** kinetics of quantum yields; Y(II): quantum yield of photochemical energy conversion; Y(NPQ): quantum yield of regulated nonphotochemical energy loss; Y(NO): quantum yield of nonregulated nonphotochemical energy loss. Results are displayed as mean ± SD, *n* = 3. The initial concentrations of glycerol in MIXO-1, MIXO-2, and MIXO-4 are 1 g L^−1^, 2 g L^−1^ and 4 g L^−1^, respectively

Several external carbons sources, including glycerol, glucose, sorbitol, trehalose and sucrose, were evaluated to promote growth of *Cylindrotheca* sp. by performing mixotrophy. The growth kinetics of *Cylindrotheca* sp. in medium with various carbon sources indicated that glycerol was the sole carbon source displaying promoting effect (Additional file 1: Fig. S1). Thus, glycerol was selected for further study to decipher the underlying mechanism. Growth kinetics in Fig. 1a showed *Cylindrotheca* sp. in all mixotrophic groups with glycerol supplementation grew faster than autotrophic cells as control. The growth rate (Table 1) of group MIXO-1, MIXO-2 and MIXO-4 in the exponential phase was increased by more than 30%, as compared to the control. Furthermore, the promoting effects of glycerol were also observed in chlorophyll a and biomass concentration (Fig. 1b and c). Although a maximum concentration of more than 1 g·L^−1^ in dry weight was achieved in all groups, it took six days for mixotrophic culture to reach the maximum concentration, as compared to a duration of 8 days in the control (Fig. 1a and c). This led to a significantly higher biomass productivity in group MIXO-2 and MIXO-4 (*p* < 0.05), which was increased by approximately 50% in comparison to the control (Table 1). Nevertheless, neither growth rate nor biomass productivity was further improved when the glycerol concentration increased from 1 g·L^−1^ to 4 g·L^−1^ (Table 1). The chlorophyll a concentration adopted a similar trend as the biomass accumulation performance (Fig. 1b). Despite a higher final chlorophyll a content in the control (Table 1), the differences between autotrophic and mixotrophic culture are not statistically significant (*p* > 0.05). Fucoxanthin serves as an accessory pigment of both photosystems, which harvests photons and transfers to light reaction centers [7]. Additionally, fucoxanthin has been considered as one of the high-value algal metabolites with various bioactivities [7]. Given its important cellular functions and biotechnology potentials, fucoxanthin yield was also quantified. The results indicated all groups, including autotrophic and mixotrophic culture, maintained a relatively similar fucoxanthin content and no significant differences were found among mixotrophic groups (Table 1). With the biomass productivity and fucoxanthin content, fucoxanthin productivity was calculated, which suggested significant increases by 29% and 41% in MIXO-2 and MIXO-4, respectively, as compared to the control (*p* < 0.05). Although fucoxanthin yield was increased from 3.316 mg L^−1^ d^−1^ to 3.630 mg L^−1^ d^−1^ by increasing the glycerol concentration from 2 g L^−1^ to 4 g L^−1^, glycerol concentration at 2 g L^−1^ was considered as the optimal concentration from a practical and economic point of view. Diatoms, performing autotrophy and mixotrophy, displayed similar ETR and quantum yields along a gradient of photosynthetic active radiation (Fig. 1d) and e). No evident depression of ETR and quantum yields of photosystem II occurred even with 4 g·L^−1^ glycerol supplementation (Fig. 1d and e). Therefore, we can conclude that mixotrophy by using glycerol can substantially stimulate biomass and fucoxanthin productivity without a reduction in pigment content and photosynthetic performance.

**Table 1:** Growth, biomass and fucoxanthin yields of autotrophic and mixotrophic *Cylindrotheca* sp.

	AUTO	MIXO-1	MIXO-2	MIXO-4
Growth rate (d^−1^)	0.284 ± 0.005	0.376 ± 0.018	0.382 ± 0.010	0.396 ± 0.010
Biomass productivity (g L^−1^ d^−1^)	0.116 ± 0.008	0.167 ± 0.020	0.176 ± 0.014	0.172 ± 0.021
Chlorophyll a content (%)	3.328 ± 0.248	2.651 ± 0.163	2.644 ± 0.120	3.006 ± 0.442
Fucoxanthin content (%)	2.227 ± 0.128	1.865 ± 0.073	1.887 ± 0.084	2.139 ± 0.312
Fucoxanthin productivity (mg L^−1^ d^−1^)	2.571 ± 0.042	3.119 ± 0.409	3.316 ± 0.133	3.630 ± 0.123

Values are displayed as average ± SD, *n* = 3. AUTO and MIXO stand for autotrophy and mixotrophy, respectively. The initial glycerol concentrations in MIXO-1, MIXO-2 and MIXO-4 are 1 g L^−1^, 2 g L^−1^ and 4 g L^−1^, respectively

### Overview of the time-series transcriptomic analysis

**Fig. 2:**
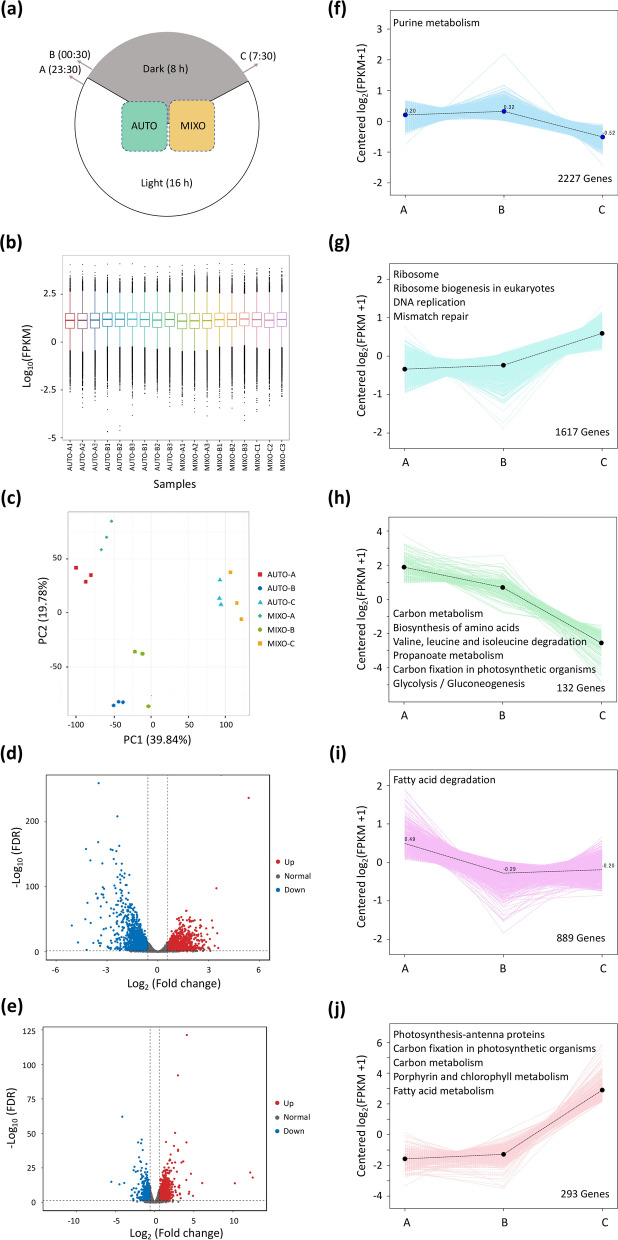
Time-series transcriptomic analysis of autotrophic and mixotrophic *Cylindrotheca* sp. highlights the transition from light into darkness. **a** Sampling timepoints from autotrophic (AUTO) and mixotrophic (MIXO) culture with biological triplicates (A: half one hour before darkness; B: half one hour after darkness; C: half one hour before light); **b** distribution of gene expression level in log_10_ (FPKM) in a box plot; FPKM: fragments per kilobase of transcript per million fragments mapped; **c** principal component analysis (PCA) of all collected samples; **d**, **e** differentially expressed genes (DEGs) from AUTO-A vs AUTO-B and AUTO-A vs MIXO-A displayed in volcano plots. Red, blue, and grey dots represent upregulated, downregulated and nonDEGs, respectively; FDR: false discovery rate; **f**–**j** representative common expression patterns of DEGs in autotrophic culture with significantly enriched KEGG pathways in each pattern noted (*q* < 0.05)

Results in Additional file 1: Fig. S1 also suggested that *Cylindrotheca* sp. could not grow in medium with any of the tested carbons sources without light by performing heterotrophy. The elucidation of light regulation on glycerol assimilation could contribute to a further improvement of the promoting effect via metabolic manipulation. Towards this end, samples from autotrophic and mixotrophic culture, were collected at three representative timepoints in the middle of exponential phase, as indicated in Fig. 2a. The time-series transcriptomic analysis, focusing on the transmission from light into darkness, can not only help understand the regulation of light on genes involved in glycerol metabolism, but also unravel the mechanism of the stimulating effect on biomass accumulation.

A total amount of 119.15 Gb clean data was generated after quality control of the raw sequencing data, with more than 5.75 Gb clean data obtained from each sample. More than 94.38% of bases in each sample possessed a quality score of no less than Q30, indicating a high accuracy. The range and distribution of gene expressions in each sample are shown in a box plot (Fig. 2b), visualizing the overall expression levels of all the samples. Principal component analysis (PCA) indicated that first and second principal component could explain approximately 60% of the variance (Fig. 2c). A grouping of the biological triplicates reflected the reproductivity within the groups, while the clear differentiation between the control and treatments indicated distinct gene expression patterns (Fig. 2c). Moreover, groups, collected at different points, were better separated than those collected in different trophic modes (Fig. 2c). Volcano plots in Fig. 2d and e offered an overall view of differentially expressed genes (DGEs) between two groups and more DEGs have been obtained between the groups collected at different timepoints than samples in different trophic modes at the same timepoint, which was consistent with the PCA results in Fig. 2c. Several bioinformatic approaches, including Kyoto Encyclopedia of Genes and Genomes (KEGG) enrichment analysis and gene set enrichment analysis (GSEA) were performed to efficiently narrow down DEGs to subsets of interests. Totally eight time-course DEG clusters in autotrophic samples were identified, five of which are shown in Fig. 2f–j with significantly enriched KEGG pathways noted (*q* < 0.05). Transcriptional levels of DEGs involved in purine metabolism peaked at B and then showed a descending trend in darkness between B and C (Fig. 2f). DEGs, associated with ribosome, ribosome biogenesis in eukaryotes, DNA replication and mismatch repair, were upregulated in the whole time-course experiment (Fig. 2g). This might indicate that samples collected at A and B were in G1 phase where preparations were made for DNA replication, while samples at point C were in S phase where DNA replication took places [29]. On the contrary, DGEs related to carbon metabolism, biosynthesis of amino acids, valine, leucine and isoleucine degradation, proponoate metabolism, carbon fixation and glycolysis/gluconeogenesis were downregulated through the whole process (Fig. 2h). Downregulation of genes in fatty acid degradation occurred mainly in the transmission period (Fig. 2i). Photosynthesis-related DEGs, in antenna proteins, carbon fixation and porphyrin and chlorophyll metabolism, together with some DEGs in carbon and fatty acid metabolisms, were maintained at a lower level between A and B but were dramatically upregulated at timepoint C even before illumination switched on again implying a potential circadian characteristic (Fig. 2j). Although it was reported algal DNA replication and cell duplication occurred during night, related changes in global transcriptome were rarely introduced [30].

### Identification of light-dependent genes in glycerol assimilation pathway

**Fig. 3:**
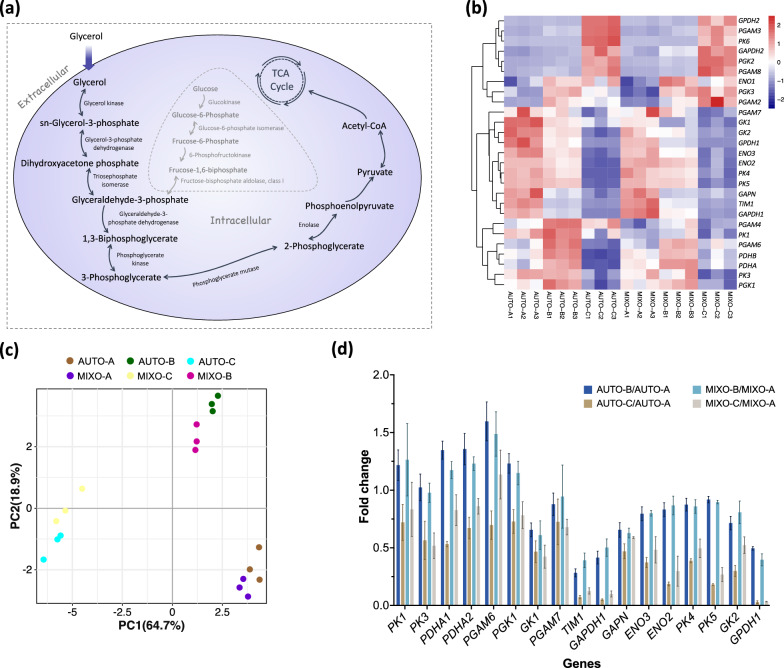
Light regulation on glycerol utilization in *Cylindrotheca* sp. **a** Proposed glycerol utilization pathway in microalgae; TCA: tricarboxylic acid; **b** changes in the transcriptional level of DEGs encoding enzymes in glycerol utilization pathway are displayed in a heatmap. GK: glycerol kinase; GPDH: glycerol-3-phosphate dehydrogenase [NAD (+)]; TIM: triosephosphate isomerase; GAPDH: glyceraldehyde-3-phosphate dehydrogenase; PGK: phosphoglycerate kinase; GAPN: glyceraldehyde-3-phosphate dehydrogenase (NADP+); PGAM: phosphoglycerate mutase; ENO: enolase; PK: pyruvate kinase; PDHA/PDHB: pyruvate dehydrogenase E1 component alpha/beta subunit; DLAT: dihydrolipoamide acetyltransferase; AUTO: autotrophy; MIXO: mixotrophy; **c** principal component analysis (PCA) for the genes involved in glycerol metabolism; **d** fold changes in gene expressions at point B and C, quantified by FPKM, compared to those at point A in each trophic mode. Analysis was carried out for biological triplicates and results are displayed as mean ± SD, *n* = 3

We further narrowed down the scope to DGEs involved in glycerol utilization, as illustrated in Fig. 3a [24, 28]. The time-course changes in the expression levels of DEGs in glycerol utilization pathway under both trophic modes are shown in a heatmap (Fig. 3b). As these processes take place in cytoplasm, isoforms present in chloroplast are excluded. PCA (Fig. 3c) indicated samples, in terms of genes related to glycerol utilization, were better separated by sampling timepoints than trophic modes, resembling the overall gene expression in Fig. 2c. DEGs in the heatmap were categorized into two groups, with one where gene expressions took an increasing trend and culminated at point C and the other one in which gene expressions were most downregulated at point C. To provide more insights into the incapability of *Cylindrotheca* sp. to use glycerol, we focused on the DEGs that were downregulated in the dark, especially during the transition from light into the darkness (between A and B) (Fig. 3d). Enzymes, including GK, GPDH and TIM are involved in glycerol assimilation [28]. The expressions of their encoding genes, including *GK1*, *GK2*, *GPDH1* and *TIM1*, showed a decreasing trend, except *GPDH2* was upregulated at point C in the dark. Among the DEGs in glycerol metabolism, *TIM1*, gene of glyceraldehyde-3-phosphate dehydrogenase (*GAPDH1*) and *GPDH1* were found as the most light-dependent genes (Fig. 3d). The expressions of *TIM1*, *GAPDH1* and *GPDH1* were reduced to 28%, 42% and 49%, respectively, after half one hour darkness (point B), as compared to those at point A under autotrophy and to 39%, 50% and 40% under mixotrophy. The suppression of the gene expressions extended to almost the end of darkness. The expressions of *TIM1*, *GAPDH1* and *GPDH1* were decreased to 7%, 5% and 3% in autotrophic culture and 13%, 10% and 3% in mixotrophic culture, respectively, at point C, as compared to point A. In contrast, the encoding genes of enzymes in glycolysis displayed mixed expression trends (Fig. 3b and d). The decrease in transcript abundances of *GK2*, *TIM1*, *GAPDH1* and *GPDH1* at point B and C, compared to those at point A in mixotrophic culture, was also confirmed by quantitative real-time PCR (qRT-PCR) results (Additional file 1: Fig. S2).

### Transcriptomic and metabolomic responses to glycerol supplementation

**Fig. 4:**
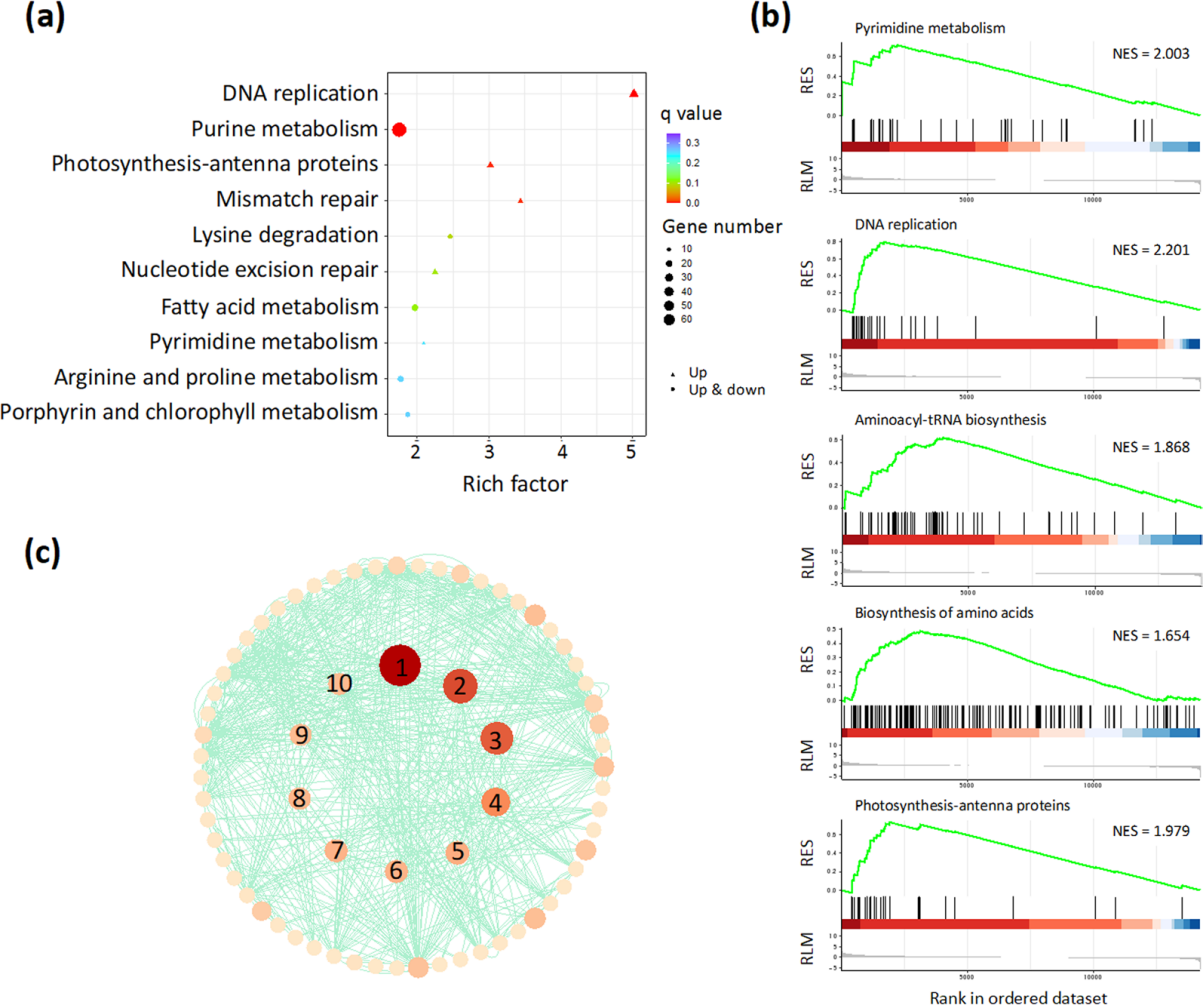
Comparative transcriptomic analysis between mixotrophy and autotrophy at point A. **a** KEGG enrichment analysis. Pathways with *q* < 0.05 are considered significantly enriched; **b** gene set enrichment analysis (GSEA). KEGG pathways with *q* < 0.05 are considered significantly enriched and corresponding normalized enrichment score (NES) is noted. RES: running enrichment score; RLM: ranked list metric; **c** protein–protein interaction network of genes in significantly enriched KEGG pathways in GSEA with top ten hub genes, evaluated by betweenness, in the center. Nos. 1–10 represent glutamate-tRNA ligase, isoleucine-tRNA ligase, anthranilate synthase, phenylalanine-tRNA ligase beta subunit, arogenate/prephenate dehydratase, aspartate aminotransferase, aspartate transaminase, tyrosine-tRNA ligase, cytoplasmic leucine-tRNA ligase and chloroplastic/mitochondrial leucine-tRNA ligase, respectively. Analysis was carried out for biological triplicates

**Fig. 5:**
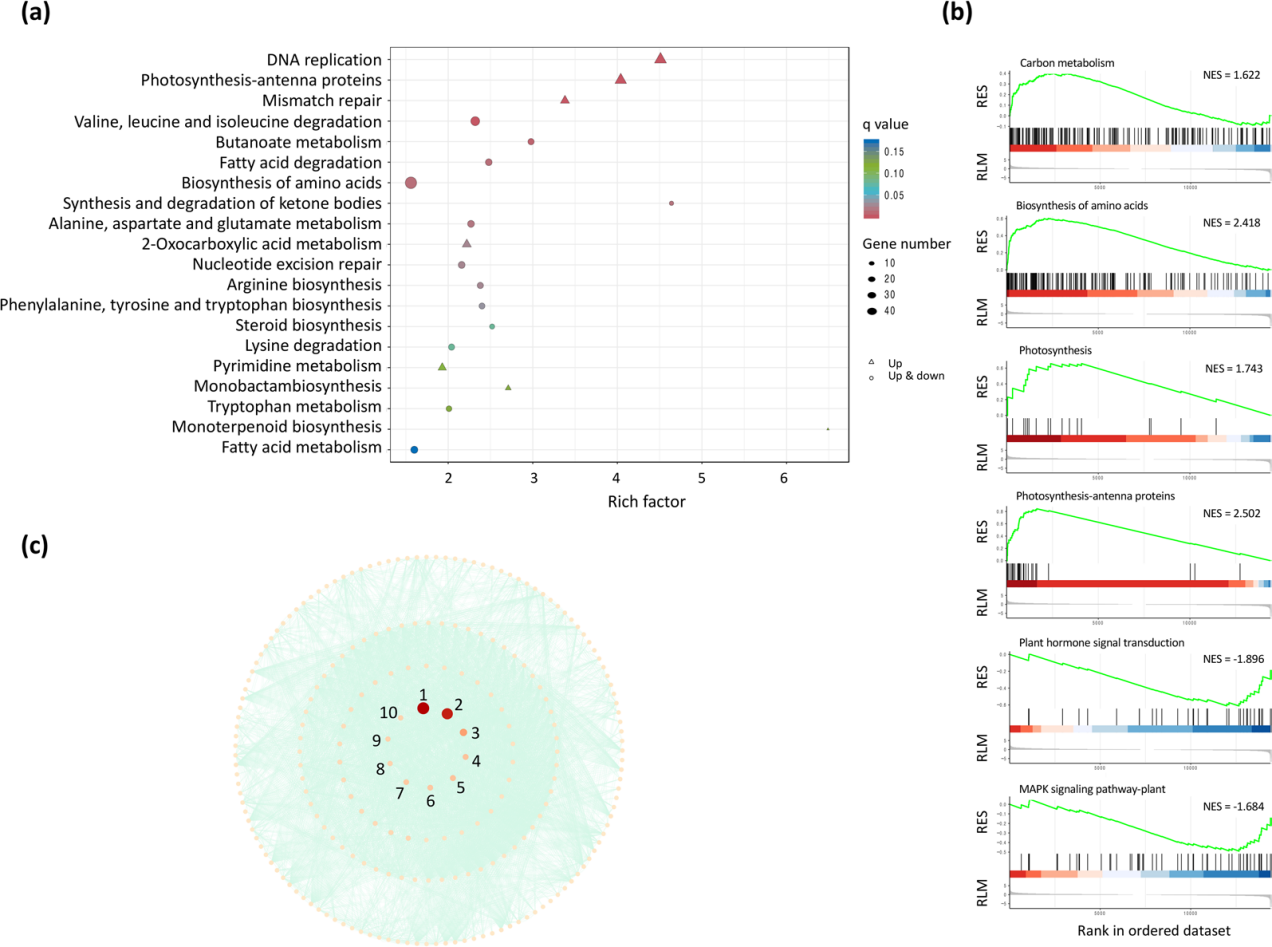
Comparative transcriptomic analysis between mixotrophy and autotrophy at point C. **a** KEGG enrichment analysis. Pathways with *q* < 0.05 are considered significantly enriched; **b** representative gene set enrichment analysis (GSEA). Pathways with *q* < 0.05 are considered significantly enriched and corresponding normalized enrichment score (NES) is noted; RES: running enrichment score; RLM: ranked list metric; **c** protein–protein interaction network of genes in significantly enriched KEGG pathways in GSEA analysis with top ten hub genes, evaluated by betweenness, in the center. Nos. 1–10 represent 26S proteasome non-ATPase regulatory subunit 11 homolog, RNA polymerase III subunit Rpc25, CDK-activating kinase assembly factor MAT1, nucleolar protein 5–2, nucleolar protein 5–2, proliferating cell nuclear antigen, 26S proteasome regulatory subunit 6B homolog, replication protein A 32-kDa subunit B, replication protein A 70-kDa DNA-binding subunit A and carbamoyl-phosphate synthase large chain. Analysis was carried out for biological triplicates

In addition to the identification of the light-dependent enzymes, the mechanism of biomass promotion by glycerol has not yet been well understood. Comparative transcriptomic and metabolomic analyses between mixotrophy and autotrophy were performed to reveal the fate of carbon flux and energy generated from TCA cycle. Bioinformatic analyses such as KEGG enrichment and GSEA were applied to identify the significantly associated pathways or gene sets at the three timepoints. At the end of illumination (point A), DEGs were significantly enriched into four pathways as shown in Fig. 4a (*q* < 0.05). The transcriptional levels of DEGs associated with DNA replication, photosynthesis-antenna proteins and mismatch repair in mixotrophy were higher than in autotrophy (Fig. 4a). Most of the DEGs at point A have been enriched into purine metabolism. Moreover, GSEA confirmed the upregulation of photosynthesis-antenna proteins and DNA replication and also revealed a significant upregulation in pyrimidine metabolism, biosynthesis of amino acids as well as aminoacyl-tRNA biosynthesis in Fig. 4b. More related pathways can be identified by GSEA, as GSEA takes all differentially expressed genes into account without an exclusion of genes with minor changes. Protein–protein interaction (PPI) network of the enzymes, encoded by the genes in significantly enriched gene sets in GSEA (*q* < 0.05), was depicted with centrality ranked by betweenness (Fig. 4c). Top ten proteins, with the highest betweenness, were listed in the central part of all proteins. All the top ten proteins (glutamate-tRNA ligase, isoleucine-tRNA ligase, anthranilate synthase, phenylalanine-tRNA ligase beta subunit, arogenate/prephenate dehydratase, aspartate aminotransferase, aspartate transaminase, tyrosine-tRNA ligase, cytoplasmic leucine-tRNA ligase and chloroplastic/mitochondrial leucine-tRNA ligase) in the central position are encoded by genes related to aminoacyl-tRNA and amino acids biosynthesis. KEGG enrichment and GSEA in Additional file 1: Figs. S3a and S3b indicated upregulation in the same pathways in mixotrophy at point B as at point A in the dark, even though genes related to glycerol uptake were downregulated. In Additional file 1: Fig. S3c, besides genes in aminoacyl-tRNA biosynthesis, genes encoding replication protein, DNA polymerase and DNA primase were among the most connected ones at point B. At point C, KEGG enrichment suggested DNA replication, photosynthesis-antenna proteins as well as mismatch repair were upregulated in mixotrophic culture (Fig. 5a). More specifically, the biosynthesis of a variety of amino acids such as lysine, arginine, valine, leucine and isoleucine were upregulated in GSEA, and valine, leucine and isoleucine degradation pathway downregulated as compared to the autotrophic culture (Fig. 5b). Transcript abundances of genes associated with carbon metabolism including glycolysis, photosynthetic carbon fixation and pentose phosphate were higher than autotrophic culture at point C. Moreover, photosynthesis-antenna proteins and photosynthetic pigments were also promoted in mixotrophy before light switched on again. The supplementation of glycerol also stimulated genes involved in DNA duplication-related processes (Additional file 1: Fig. S4). It is worth noting the expression of some genes in plant hormone signal transduction and MAPK signaling pathways were suppressed in medium with glycerol supplementation (Fig. 5b). PPI network showed 26S proteasome non-ATPase regulatory subunit 11 homolog, RNA polymerase III subunit Rpc25 and CDK-activating kinase assembly factor MAT1 were found to be hub genes (Fig. 5c).

**Fig. 6:**
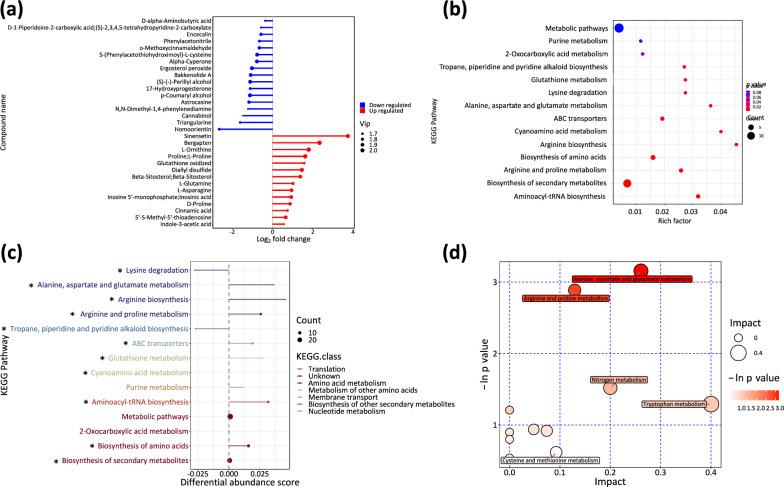
Comparative metabolomic analysis between mixotrophy and autotrophy in daytime. **a** Differentially expressed metabolites with *p* < 0.05 and VIP > 1; **b** KEGG enrichment analysis; **c** differential abundance score indicates a general trend of a pathway. “*” represents significance (*p* < 0.05); **d** pathway impacts can be assessed with topological analysis in which pathways with higher impact and significance are larger in size of bubble and darker in color. Analysis was carried out for biological triplicates

Changes in metabolome of samples at the end of exponential phases during the daytime from mixotrophic culture, as compared to the autotrophic mode, were measured with LC–MS in both positive and negative modes to further explain the promoting effect of glycerol on biomass accumulation. PCA and orthogonal projections to latent structures-discriminant analysis (OPLS-DA) indicated a significant separation of the two groups (Additional file 1: Fig. S5). Totally 31 differentially expressed metabolites (DEMs) were identified between mixotrophy and autotrophy (control), as shown in Fig. 6a. Among the DEMs, 14 were significantly upregulated and 17 downregulated in mixotrophy, as compared to the control. They belong to several classes including phytohormone, flavonoids, coumarins, phenylpropanoids, amino acid, nucleotide, benzene, carboxylic acids, cinnamic acids and derivatives. The DEMs were significantly enriched in 11 KEGG pathways including biosynthesis of aminoacyl-tRNA, secondary metabolites and amino acids metabolism (Fig. 6b**)**. Differential abundance score (Fig. 6c), providing an overall view of changes in all metabolites in a specific pathway, manifested amino acid metabolisms were significantly upregulated, together with aminoacyl-tRNA and ABC transporters. This is consistent the transcriptomic results above. Lysine degradation and tropane, piperidine and pyridine alkaloid biosynthesis were significantly downregulated. An integrated enrichment and topology analysis of the involved KEGG pathways enabled a further identification of alanine, aspartate and glutamate metabolism (*p* < 0.05; impact factor = 0.26) as one with the highest impact between mixotrophy and autotrophy (Fig. 6d). Thus, it can be concluded that metabolisms of amino acids and aminoacyl-tRNA in mixotrophic culture were maintained at a higher level than in autotrophic culture in exponential phase, though samples for analysis collected at different timepoints within diurnal cycles.

## Discussion

Of the tested carbon sources, only glycerol showed a clear and significant promoting effect on the growth of mixotrophic *Cylindrotheca* sp. and *Cylindrotheca* sp. cannot perform heterotrophy on any of the carbon sources without the presence of light (Additional file 1: Fig. S1). External carbon sources were applied to *Cylindrotheca* sp. cultivation in a previous study, in which no stimulatory effect was shown in mixotrophy with glycerol and sodium acetate [31]. Thus, the leverage of glycerol as a supplementation of carbon can be a species-specific characteristic and it does not apply to all *Cylindrotheca*. In addition to glycerol, glucose was frequently used in mixotrophy, due to its high energy content per mol [28, 32]. Results indicated glucose can been metabolized by *P. tricornutum* by performing mixotrophy, but it was not used in the dark via an isotope labeling approach [33]. *P. tricornutum* showed flexibility in organic carbon sources and the promoting effects on *P. tricornutum* growth was examined [34, 35]. The incapability of *Cylindrotheca* sp. to metabolize glucose (Fig. 3a) can be explained by missing transport systems in its genome, while glycerol can enter cells by direct diffusion. Nevertheless, heterologous expression of a glucose transporter in *P. tricornutum* could not result in growth in the dark [36]. Hence, to enrich the types of available carbon sources for *Cylindrotheca* sp. via glycolytic and pentose phosphate pathways, a proper heterologous transport system can be introduced in next step. In the present study, the promoting effect of glycerol on biomass productivity is independent of glycerol concentration ranging from 1 g L^−1^ to 4 g L^−1^, which is consistent with results in a batch cultivation of *P. tricornutum* with glycerol, revealing a possible upper limit of glycerol utilization [34]. Loss of genes involved in photosynthesis in heterotrophic *Nitzschia* sp. was reported previously [37]. Therefore, mixotrophy is particularly advantageous in the production of photosynthesis-related metabolites, even though both mixotrophy and heterotrophy are considered as alternatives to autotrophy to enhance biomass yield. However, the impact of organic carbon on photosynthesis and photosynthetic pigments varies among different species. Recent study revealed that mixotrophic cultivation with glucose could bypass the function of photosynthesis to some extent, evidenced by a significantly reduced NPQ in *Chromochloris zofingiensis* [38]. This inhibitory effect of organic carbon source on photosynthesis was also observed in *P. tricornutum* [25]. Despite the improvement in growth, photosynthetic performance, such as oxygen evolution rate, maximum quantum yield (*F*_*v*_/*F*_*m*_) and ETR, of *P. tricornutum* grown in medium with supplementation of glycerol, glucose and acetate was substantially reduced, as compared to autotrophic culture [25]. However, *P. tricornutum*, grown with glycerol, accumulated more chlorophyll and carotenoids than autotrophic cells at 190 µmol m^−2^ s^−1^ continuous light, indicating a possible light protection function under high light intensity [34]. In contrast, no clear inhibition was found in the groups with glycerol supplementation in this study, compared to the control, in terms of quantum yields of photochemical energy conversion (Y(II)) and regulated nonphotochemical energy loss (Y(NPQ)), ETR and chlorophyll a content. In our previous study, fucoxanthin yields of four diatoms species, including *Cylindrotheca closterium*, *Amphora* sp., *P. tricornutum*, and *Thalassiosira weissflogii*, were assessed in similar PBRs which were also used in this study [16]. Fucoxanthin productivity of the four species ranged from 0.37 to 1.08 mg L^−1^ d^−1^ in 2 × F/2 medium [16]. Of particular interest is in the current work the fucoxanthin productivity of *Cylindrotheca* sp. in both autotrophic and mixotrophic culture (2.57–3.63 mg L^−1^ d^−1^), is remarkably higher than our previous results. This can be attributed to more sufficient nutrients (modified 8 × F/2 medium) supply, axenic culture and supplementation of glycerol, in addition to a different strain in this study.

Despite the wide applications of glycerol in improving biomass and bioproducts yields in microalgae, the regulation of glycerol metabolism and the mechanism underlying promoting effects are still insufficiently understood. In the present study, comparative transcriptome and metabolome between autotrophic and mixotrophic culture were performed for samples collected at different timepoints within diurnal cycles to gain some novel insights on a molecular level. Based on the results, the inability of *Cylindrotheca* to use glycerol in darkness could be explained either by the slowdown of the whole pathway induced by a reduced phosphorylation rate catalyzed by GK, as the rate-limiting enzyme, or a concomitant downregulation of GK, GPDH and TIM involved in glycerol assimilation. To increase the lipid productivity, endogenous GK was overexpressed in *Fistulifera solaris* to enhance glycerol metabolism in mixotrophic culture with glycerol supplementation [39]. Transformants, with higher GK-expression level, exhibited a slightly higher lipid yield (12%), but this was not applied to all transformants [39]. The unsatisfactory results could be possibly interpreted by its light-dependent property, which is in line with the results in the present study, and during the daytime GK may not be a rate-limiting enzyme in glycerol metabolism. For this reason, heterologous expression of light-independent enzymes in glycerol metabolism from other organism may contribute to the further improvement of biomass accumulation. Relatively better-established glycerol pathways in bacteria, hosting a similar glycerol assimilation pathway as diatom, may offer more hints [40, 41]. Heterologous expression of GK from *E. coli* in *Ralstonia eutropha*, with important biotechnology potentials, achieved a higher glycerol uptake rate, compared to inefficient uptake in wild type due to a lower kination activity of GK [42]. The ideal situation for diatom mixotrophy will be a constant uptake of glycerol independent of light. In addition to GK, the bacteria-derived glycerol pathway provides an alternative for diatom or other microalgae to bypass the regulation by light. *Lactobacillus panis*, with a complete glycerol oxidative pathway including glycerol facilitator, GK, GPDH and TIM from *E. coli*, was able to directly metabolize glycerol in the medium [43]. Future studies of establishing a heterologous glycerol uptake pathway will be feasible with synthetic biology approaches.

Although enzymes in glycolysis and gluconeogenesis could catalyze reversible reactions, studies suggested that the assimilation of glycerol could suppress steps in gluconeogenesis and pentose phosphate pathways due to a reduced activity of glucose-6-phosphate isomerase [44]. As recently was disclosed, mixotrophy of *Chromochloris zofingiensis* with glucose promoted biomass accumulation by employing a different strategy, in which intermediates of glycolysis could directly enter the Calvin–Benson–Bassham (CBB) cycle and promote cellular growth by bypassing ribulose-1,5-bisphosphate carboxylase/oxygenase (Rubisco) [38]. In this study, genes encoding key enzymes in CBB cycle and light reactions of photosynthesis were upregulated in mixotrophy at point A, together with the C4 carboxylase, phosphoenolpyruvate carboxylase. 6-Phosphofructokinase, catalyzing the conversion from fructose-6-phosphate to fructose-1,6-bisphosphate, is an irreversible step in glycolysis (Fig. 3a). Its upregulation in mixotrophic culture might indicate the supplementation of glycerol, different from glucose, exerted an indirect impact on the upregulation of photosynthesis-related genes in *Cylindrotheca* sp. However, the unaffected quantum parameters in mixotrophic *Cylindrotheca* sp. culture might be due to another bottleneck, for example Rubisco, in photosynthesis.

At point C before light switched on again, more pathways were stimulated in mixotrophy than at the other two timepoints possibly due to a circadian characteristic. As reflected in Fig. 2g, the expression of genes related to DNA replication in autotrophic culture increased in the dark between point B and C, while in mixotrophic culture genes involved in DNA replication and ribosome biogenesis were further promoted. Based on the results in Figs. 3, 4, 5, 6, it could be inferred although genes related to glycerol assimilation were downregulated, the impact of glycerol on cellular metabolisms extended through darkness. Pyrimidine, DNA replication, amino acids, aminoacyl-tRNA biosynthesis as well as antenna proteins were upregulated throughout the time-series experiment in mixotrophic culture as compared to control. The upregulation of amino acids and aminoacyl-tRNA metabolisms were also confirmed by metabolomic results with samples in daytime. With the transcriptomic and metabolic analyses, it can be concluded metabolic flux from TCA cycle in cells grown with glycerol supplementation was guided more into metabolisms of pyrimidine, amino acids and aminoacyl-tRNA, which was further used for DNA and protein production. Therefore, this could account for the biomass promotion in *Cylindrotheca* sp. In contrast, it was also reported that the supplementation of glycerol could significantly increase the growth of *P. tricornutum* and TAG accumulation by upregulating genes involved in central carbon, carbon storage and lipid metabolisms [24]. Additionally, metabolomic analysis indicated a significant downregulation of several amino acids such as valine, alanine, leucine and guanidine in *P. tricornutum* [24]. This discrepancy could possibly be explained by the fact that the *P. tricornutum* was collected in its stationary phase when nutrients were insufficient. On the contrary, *Cylindrotheca* sp. collected in this study were in its exponential phase. Therefore, it can be further speculated the promoting effects by glycerol can be affected by the stages of cells. Transcriptional level of genes in amino acids biosynthesis, in *Chlorella sorokiniana* grown with acetate, displayed mixed patterns, in which genes associated with some amino acid metabolisms were upregulated and others downregulated [45]. Hence, the complex mechanisms underlying biomass promotion varies among different carbon sources.

## Conclusions

*Cylindrotheca* sp. of wide biotechnology applications, grown mixotrophically with glycerol supplementation, showed significantly higher biomass and fucoxanthin yields than autotrophic culture. Transcriptomic analysis revealed *GPDH1*, *TIM1* and *GAPDH1*, involved in glycerol utilization pathway, exhibited strong light-dependent characteristics. Replacement of light-dependent genes offers an alternative to further improve biomass productivity in mixotrophic culture. Comparative transcriptomic and metabolomic analyses proved the metabolisms of amino acids and aminoacyl-tRNA were upregulated in mixotrophic culture at different timepoints within diurnal cycles. These findings could account for the biomass promotion in *Cylindrotheca* sp. and serve as a foundation for further improvement.

## Methods

### Algae and stock culturing

*Cylindrotheca* sp. was a curtesy of the Lab of Applied Microalgae Biology, Ocean University of China, Qingdao, China. Stock culture was maintained in modified and sterilized 8 × F/2 medium, in which eight times nutrients and 12 times of vitamin mix of F/2 medium was added [46]. Stock solution was placed under 22 ± 1 °C, light intensity of 20 µmol m^−2^ s^−1^ and regime of 16 h/8 h light/darkness. Axenic culture was obtained by streaking algae on 2 × F/2 solid medium with agar (1%, v/v), neomycin, gentamycin and kanamycin (150 mg L^−1^).

### Batch cultivation of* Cylindrotheca* sp.

*Cylindrotheca* sp. was pre-cultivated in the modified and sterilized 8 × F/2 medium without and with glycerol, respectively, for nine days before a batch cultivation under 22 ± 1 °C, light intensity of 100 μmol m^−2^ s^−1^ and regime of 16 h/8 h light/darkness. Algae were subsequently inoculated into the modified and sterilized 8 × F/2 medium without glycerol as control and medium with a gradient of glycerol (1 g L^−1^, 2 g L^−1^ and 4 g L^−1^) by performing mixotrophy under the same environmental conditions. Illumination was provided with LED light (Opple, T5-8W, China) and light intensity was measured on the surface of PBRs facing towards the light source by a photometer (LI-COR, LI250A, USA). Batch cultivation was carried out in lab-made bottle PBRs containing 700 mL medium in each one. Aeration was provided by air pumps (GRECH, CQ-100, China) constantly and the air was filtered by two 0.22 µm filters (JINTENG, PES, China). Biological triplicates have been prepared for all treatments. Cultivation of autotrophic and mixotrophic *Cylindrotheca* sp. last eight and six days, respectively, to keep both autotrophic and mixotrophic diatoms at the same stage (late exponential phase), according to our preliminary results.

### Characterization of growth, pigments and photosynthetic performance

Growth performance was evaluated by measuring optical density at 750 nm daily with a spectrophotometer (UNICO, UV2350, China) and biomass concentration in dry weight was measured the other day. A gravitational method was applied to quantify the biomass concentration. An aliquot volume of algal solution was filtered onto a pre-weighed 0.22-μm glass fiber filter (Xingyacailiao, Jinjing, China) and rinsed with twice volume of de-ionized water. Filters loaded with biomass were dried in a 65 °C oven overnight. Pigments were extracted by vortex assisted by glass beads with methanol as solvent. Chlorophyll a concentration was quantified with the equation reported previously by reading absorbance at 653 and 666 nm [47]. Fucoxanthin was quantified with a HPLC (SHIMADZU, LC_2030C 3D, Japan) equipped with a reverse phase column (YMC, Carotenoid 5 µm, Japan) and a commercial standard (SolarBio, Fucoxanthin, China). Absorbance was recorded at 450 nm and elution program was previously reported by Huang et al. with minor modifications [48]. In this study, organic solvent (methanol/isopropanol, 4:1) in 100% was maintained from 10 to 15 min and the whole procedure finished in 20 min. The partition of absorbed excitation energy in photosystem II was estimated following the reported equations [49, 50]. Kinetics of Y(II), Y(NPQ), quantum yield of nonregulated nonphotochemical energy loss (Y(NO)) and ETR, together with *F*_*v*_/*F*_*m*_, were measured by a PAM-fluorometry (Heinz Walz GmbH, PAM2500, Germany) for all biological triplicates in each group. Cells, collected at day four within the exponential phase, were subject to 15 min dark adaptation before the quantum yields and ETR analysis. Significances have been evaluated with ANOVA (single factor) and Student’s *t*-test with an alpha value of 0.05 with Office Excel.

### Time-series transcriptomic analysis

Autotrophic and mixotrophic (2 g L^−1^ glycerol) batch cultivation with biological triplicates was repeated to accumulate enough biomass for transcriptomic and metabolomic analyses, respectively. Samples, for time-series transcriptomic analysis, were collected between day three and four at three timepoints emphasizing the transmission from light into darkness (Fig. 2a). Samples were harvested by centrifugation (Eppendorf, 5804 R, Germany) at 3000*g* for 6 min and immediately frozen in liquid nitrogen for 1 min before storage in – 80 °C freezer. RNA sequencing and transcriptomic analysis were performed by Biomarker Technologies for samples collected at three timepoints with biological triplicates. Total RNA was extracted with a kit (TIANGEN, DP411, China) and the purity and integrity of the extracted RNA were evaluated by a Nanodrop (Thermo Fisher Scientific, Nanodrop 2000, USA) and nucleic acid analyzer (Perkin-Elmer, LabChip GX, USA), respectively. After library control, RNA sequencing was performed with Illumina sequencing platforms. HISAT2 and StringTie were used for mapping RNA-seq reads to the reference *Cylindrotheca* genome (data in preparation) and assembly of mapped RNA-seq, respectively. Fragments per kilobase of transcript per million fragments mapped (FPKM) was applied to quantify the expression level of a gene or transcript by StringTie using maximum flow algorithm. FPKM equals to cDNA fragments divided by mapped fragments (millions) and transcript length (kb). Genes with fold change > 1.5 times and false discovery rate (FDR) < 0.05 were assigned as DEGs. DEGs were annotated in multiple databases. PCA and volcano plots were prepared with R packages (ggplot2 and cluster). DEGs were enriched in KEGG pathways with R package (ClusterProfiler). In contrary to a pre-set threshold on fold change, GSEA, taking minor alterations in gene expression into account, was performed with R package (ClusterProfiler). PPI network of genes, in significant enriched KEGG pathways (*q* < 0.05) in GSEA, was generated with String and visualized with Cytoscape.

### Quantitative real-time PCR

qRT-PCR was carried out to confirm the changes in the expression levels of the light-dependent genes in mixotrophic culture at the three timepoints. Total RNA extraction and subsequent reverse transcription were carried out with a plant RNA kit (Omega Bio-tek, R6827-01, USA) and a reverse transcription kit (Accurate Biotechnology, AG11728, China), respectively. Gene-specific primers used in this experiment are listed in Additional file 1: Table S1. Concentration of the isolated RNA was measured by a Nanodrop (Thermo Fisher Scientific, 2000c, USA) and its quality was assessed by gel electrophoresis. qRT-PCR reactions were performed on a real-time PCR system (Analytik Jena, qTOWER^3^, Germany) using PerfectStart Green qPCR SuperMix (TransGen Biotech, AQ601-02, China) with *ACTIN* as a reference gene. All procedures referred to protocols in the kits. Relative expression level for the genes was calculated with 2^–∆∆CT^ in biological triplicates and technical triplicates were prepared for each biological replicate.

### Metabolomic analysis

Diatoms were collected in the daytime at the end of exponential phase from autotrophic and mixotrophic culture with biological triplicates for metabolomic analysis. Metabolites extraction, UHPLC–MS analysis, annotation and data preprocessing were carried out by Shanghai Biotree Biomedical Technology co., Ltd. Significance was evaluated by p value of Student’s *t*-test and variable importance in the projection (VIP) of OPLS-DA. DEMs were defined by *p* < 0.05 and VIP > 1. PCA and OPLS-DA of metabolites were performed with SIMCA. DEMs were shown in a matchstick plot (Fig. 6a). Differential abundance score was calculated by the ratio of DEM to all metabolites in a specific pathway. Impacts of pathways, shown in a bubble plot, were calculated with topological analysis. Matchstick, KEGG enrichment of DEMs, differential abundance score and bubble plots were prepared with R packages (ggplot2 and KEGGgraph).

## Supplementary Information

**Additional file 1: Figure S1.** Evaluation of the promoting effects of different organic carbon sources on growth of *Cylindrotheca* sp. withand withoutthe presence of light. Culture was placed under 22 ± 1 °C, light intensity of 80 μmol m^−2^ s^−1^ and regime of 16 h/8 h light/darkness. Results are displayed as mean ± SD, *n* = 2. **Figure S2.** Changes in relative expression level of light-dependent genes in mixotrophic culture. Data were shown as mean ± SD, *n* = 9. GK: glycerol kinase; GPDH: glycerol-3-phosphate dehydrogenase [NAD]; TIM: triosephosphate isomerase; GAPDH: glyceraldehyde-3-phosphate dehydrogenase. **Figure S3.** Comparative transcriptomic analysis between mixotrophy and autotrophy at point B.KEGG enrichment analysis. Pathways with *q* < 0.05 are considered significantly enriched;Gene set enrichment analysis. KEGG pathways with *q* < 0.05 are considered significantly enriched and corresponding normalized enrichment scoreis noted; RES: running enrichment score; RLM: ranked list metric;Protein–protein interaction network of genes in significantly enriched KEGG pathways in GSEA analysis with top ten hub genes, evaluated by betweenness, in the center. Nos. 1–10 represent glutamate-tRNA ligase, isoleucine-tRNA ligase, phenylalanine-tRNA ligase beta subunit, tyrosine-tRNA ligase, replication protein A 32 kDa subunit B, cytoplasmic leucine-tRNA ligase, chloroplastic/mitochondrial leucine-tRNA ligase, replication protein A 70 kDa DNA-binding subunit A, DNA polymerase epsilon catalytic subunit A and DNA primase large subunit. Analysis was carried out for biological triplicates. **Figure S4.** The rest gene set enrichment analysis results between mixotrophy and autotrophy at pion C. Pathways with *q* < 0.05 are considered significantly enriched and corresponding normalized enrichment scoreis noted. Analysis was carried out for biological triplicates. RES: running enrichment score; RLM: ranked list metric. **Figure S5.** Orthogonal projections to latent structures-discriminant analysis and three-dimensional principal component analysis of metabolome in mixotrophy and autotrophy. Red dots and blue squares indicate biological triplicates of autotrophyas control and mixotrophy, respectively. **Table S1.** Primers in quantitative real-time PCR.

## Data Availability

The raw sequence data reported in this paper have been deposited in the Genome Sequence Archive (https://ngdc.cncb.ac.cn/gsa) in National Genomics Data Center, China National Center for Bioinformation/Beijing Institute of Genomics, Chinese Academy of Sciences, under accession CRA011018.

## Abbreviations

EPA: Eicosapentaenoic acid
NPQ: Nonphotochemical quenching
ETR: Electron transport rate
GK: Glycerol kinase
GPDH: Glycerol-3-phosphate dehydrogenase [NAD (+)]
TIM: Triosephosphate isomerase
TCA: Tricarboxylic acid
PBR: Photobioreactor
PCA: Principal component analysis
DGE: Differentially expressed gene
KEGG: Kyoto Encyclopedia of Genes and Genomes
GSEA: Gene set enrichment analysis
GAPDH: Glyceraldehyde-3-phosphate dehydrogenase
PPI: Protein–protein interaction
OPLS-DA: Orthogonal projections to latent structures-discriminant analysis
DEM: Differentially expressed metabolites
*F*_*v*_/*F*_*m*_: Maximum quantum yield
Y(II): Quantum yield of photochemical energy conversion
Y(NPQ): Quantum yield of regulated nonphotochemical energy loss
CBB: Calvin–Benson–Bassham
Rubisco: Ribulose-1,5-bisphosphate carboxylase/oxygenase
Y(NO): Quantum yield of nonregulated nonphotochemical energy loss
FPKM: Fragments per kilobase of transcript per million fragments mapped
FDR: False discovery rate
VIP: Variable importance in the projection
AUTO: Autotrophy
MIXO: Mixotrophy
PGK: Phosphoglycerate kinase
GAPN: Glyceraldehyde-3-phosphate dehydrogenase (NADP +)
PGAM: Phosphoglycerate mutase
ENO: Enolase
PK: Pyruvate kinase
PDHA/PDHB: Pyruvate dehydrogenase E1 component alpha/beta subunit
DLAT: Dihydrolipoamide acetyltransferase
NES: Normalized enrichment score
RES: Running enrichment score
RLM: Ranked list metric
