# Supplementary material for: Transcriptomic and metabolomic analyses revealed regulation mechanism of mixotrophic Cylindrotheca sp. glycerol utilization and biomass promotion
Source: Biotechnol Biofuels Bioprod. 2023 May 19;16:84. doi: 10.1186/s13068-023-02338-8 (PMC10199484; doi:10.1186/s13068-023-02338-8)
Supplement: Supplementary file 1 — Additional file 1: Figure S1. Evaluation of the promoting effects of different organic carbon sources on growth of Cylindrotheca sp. withand withoutthe presence of light. Culture was placed under 22 ± 1 °C, light intensity of 80 μmol m−2 s−1 and regime of 16 h/8 h light/darkness. Results are displayed as mean ± SD, n = 2. Figure S2. Changes in relative expression level of light-dependent genes in mixotrophic culture. Data were shown as mean ± SD, n = 9. GK: glycerol kinase; GPDH: glycerol-3-phosphate dehydrogenase [NAD]; TIM: triosephosphate isomerase; GAPDH: glyceraldehyde-3-phosphate dehydrogenase. Figure S3. Comparative transcriptomic analysis between mixotrophy and autotrophy at point B.KEGG enrichment analysis. Pathways with q < 0.05 are considered significantly enriched;Gene set enrichment analysis. KEGG pathways with q < 0.05 are considered significantly enriched and corresponding normalized enrichment scoreis noted; RES: running enrichment score; RLM: ranked list metric;Protein–protein interaction network of genes in significantly enriched KEGG pathways in GSEA analysis with top ten hub genes, evaluated by betweenness, in the center. Nos. 1–10 represent glutamate-tRNA ligase, isoleucine-tRNA ligase, phenylalanine-tRNA ligase beta subunit, tyrosine-tRNA ligase, replication protein A 32 kDa subunit B, cytoplasmic leucine-tRNA ligase, chloroplastic/mitochondrial leucine-tRNA ligase, replication protein A 70 kDa DNA-binding subunit A, DNA polymerase epsilon catalytic subunit A and DNA primase large subunit. Analysis was carried out for biological triplicates. Figure S4. The rest gene set enrichment analysis results between mixotrophy and autotrophy at pion C. Pathways with q < 0.05 are considered significantly enriched and corresponding normalized enrichment scoreis noted. Analysis was carried out for biological triplicates. RES: running enrichment score; RLM: ranked list metric. Figure S5. Orthogonal projections to latent structures-discriminant analysis and th [file 13068_2023_2338_MOESM1_ESM.docx]

**Supplementary information**

**Figure S1**

**Figure S2**

**Figure S3**

**Figure S4**

**Figure S5**

**Table S1 Primers in quantitative real-time PCR**

| **Gene** | **Forward primer** | **Reverse Primer** |
| --- | --- | --- |
| *GK2* | AGGAAGACTCGTTGCCCAAG | ACGGCTTTGGATCCCACATT |
| *TIM1* | TGCCCGTCGTTACCTGATTG | TTGGGCACCAACCTCAATGT |
| *GPDH1* | GGTCCATGAGGAGCAAGTGG | CTTGACAGAAAGGCATGCGG |
| *GAPDH1* | CGACTACGCCGCCTATCAAT | GACACCAGTTGACTCGCAGA |
| *ACTIN (Reference)* | TGTCCCGAGGTCCTATTCCA | CCTCCTTGGACATGCGTTCA |
